# Human amniotic membrane graft removal and histopathologic evaluation following closure of a full thickness macular hole

**DOI:** 10.1016/j.ajoc.2024.102239

**Published:** 2024-12-24

**Authors:** Hares Patel, Kayla Swiatek, Marie Rivera-Zengotita, Jinghua Chen

**Affiliations:** a University of Florida, Department of Ophthalmology, USA; b University of Florida, Department of Pathology, Immunology, and Laboratory Medicine, USA

**Keywords:** Human amniotic membrane graft, Amniotic membrane removal, Macular hole, Retinal detachment

## Abstract

**Purpose:**

Human amniotic membrane (hAM) grafts have been used to close persistent macular holes in recent years. The results from these surgeries are promising with improved closure rate and vision. However, there is lack of data for what happens to these membranes and how long the tissue should remain inside the patient's eyes. We report a case of hAM graft removal 6 months after closure of a persistent macular hole and the histopathology result of the removed hAM graft.

**Observations:**

This is a report of a 57-year-old male patient that presented with hand motion vision, bullous, macula off rhegmatogenous retinal detachment in his right eye with multiple retinal holes and tears. Patient had 25G pars plana vitrectomy (PPV) with membrane peel and perfluoro-n-octane (PFO), silicone oil (SO) tamponade. After the first surgery, patient's retina reattached with a full thickness macular hole. Four months later, patient underwent PPV, SO removal, membrane peel and 20 % gas sulfur hexafluoride (SF6) tamponade. After the second surgery, the patient's macular hole remained open. Three months after the second surgery, we performed the third surgery with placement of dehydrated human amniotic/chorionic membrane (DHACM AMNIOFIX) over the macular hole and SO tamponade. Follow-up optical coherence tomography (OCT) showed the dehydrated amniotic/chorionic membrane (DHACM) integrated into the inner retina and the macular hole closed. However, the patient experienced obstruction of his central vision due to the graft. Six months after the third surgery, we removed the patient's silicone oil and the DHACM. After DHACM removal, the patient's vision improved to 20/80 and the OCT showed that the macular hole remained closed with restoration of macular layers. The histopathology examination of the removed DHACM did not show retinal tissue attachment.

**Conclusions and importance:**

This case shows the efficacy of dehydrated human amniotic membrane (hAM) graft to close a persistent full thickness macular hole and restore retinal layers. OCT imaging, prior to hAM removal, noted the hAM integration into the inner retina. In addition, we show that the graft can be removed after 6 months without obvious damage to the retina.

## 1 Introduction

Human amniotic membrane (hAM) grafts have been used to close macular holes refractory to para plana vitrectomy and internal limiting membrane peeling. In recent years, there have been several case reports for the technique and use of hAM grafts for macular holes.^1^, ^2^, ^3^, ^4^, ^5^ The results from these surgeries are promising with improved closure rate and vision. However, there is lack of data for what happens to these membranes and how long the tissue should remain implanted in the patient's eye. We report a case of hAM graft removal 6 months after closure of a persistent macular hole with optical coherence topography (OCT) and histopathology evaluation (see Table 1).

## 2 Case report

### 2.1 History of presenting illness and hospital course

**Fig. 1:**
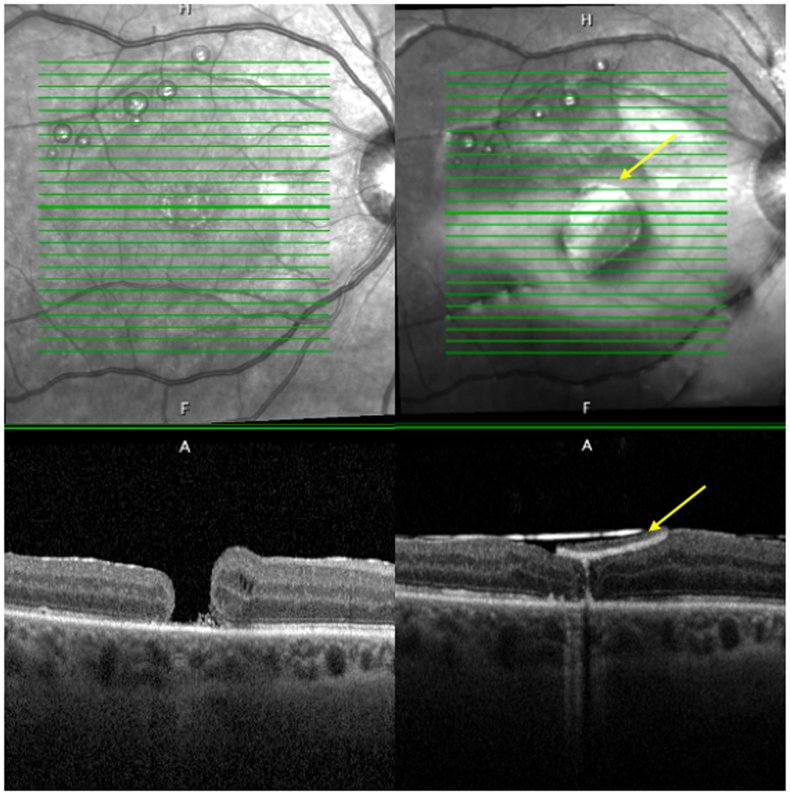
SD-OCT shows right eye pre (left photo) and post amniotic membrane graft surgery (right photo). Yellow arrow pointing to amniotic graft. Post surgery OCT showing integration of graft into inner retinal layers. Subretinal bubbles of perfluorocarbon can be seen in the superior macula of both images. (For interpretation of the references to colour in this figure legend, the reader is referred to the Web version of this article.)

**Fig. 2:**
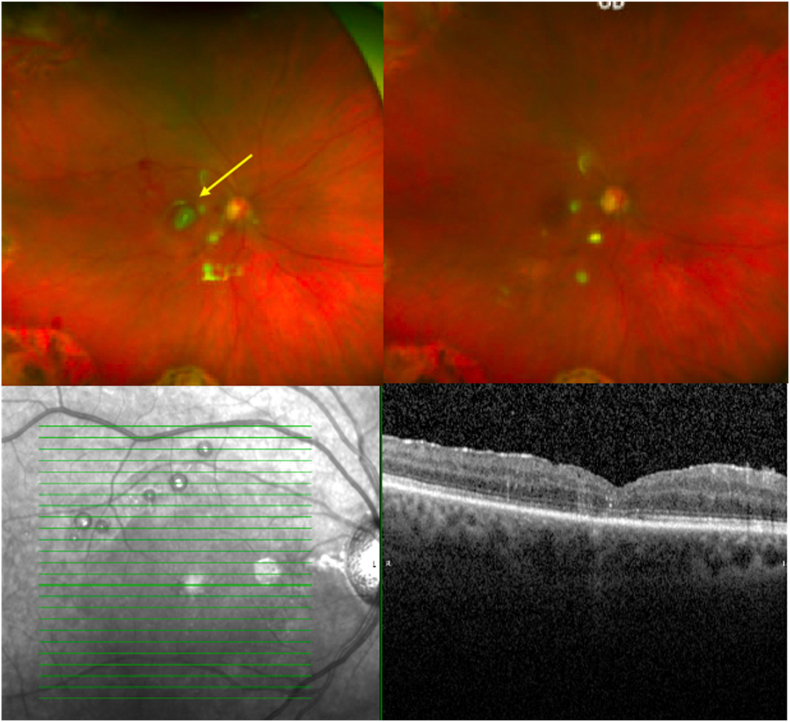
Color fundus photos show right eye pre (top left) and post amniotic membrane removal (top right). SD-OCT shows right eye macular hole remains closed after amniotic membrane removal (bottom photo). Subretinal bubbles of perfluorocarbon in the superior macula seen in both images. (For interpretation of the references to colour in this figure legend, the reader is referred to the Web version of this article.)

57-year-old male was referred to our clinic for rhegmatogenous retinal detachment in his right eye. He presented with hand motion vision, bullous, macula off rhegmatogenous retinal detachment in his right eye with 10 periphery retinal holes and tears. Patient was pseudophakic in the right eye, secondary to right eye trauma years prior. 25G Vitrectomy with membrane peel, perfluoro-n-octane (PFO) and 1000 silicone oil (SO) was performed. One month after surgery, his retina reattached with a full thickness macular hole on optic coherence tomography scan (OCT). His vision was 20/400. Three months after initial surgery, 23G vitrectomy, silicone oil removal, brilliant blue assisted internal limiting membrane (ILM) peel, fluid-Air exchange and 20 % sulfur hexafluoride (SF6) gas tamponade was performed to repair the macular hole. One month after his second surgery, the macular hole was still open (Fig. 1), VA was 20/60 eccentrically. Patient complained of extreme metamorphopsia and a central scotoma. After discussion with patient a decision was made to use (human amniotic membrane) hAM graft to assist the closure of macular hole. Three months after his second surgery, a 25G vitrectomy, fluid-air exchange, dehydrated human amnion/chorion membrane graft application (DHACM AMNIOFIX®), and 1000 silicone oil tamponade was performed. The size of the graft was 2mm in diameter. This size was determined by the smallest available trephine cutting tool. The graft was placed over the hole with the chorionic membrane side down towards the retina, the amniotic membrane side facing the vitreous cavity. Healon OVD (ophthalmic viscosurgical device) was place on top of the graft. One month after his third surgery, the patient's retina remained attached and the macular hole close. However, his vision was 20/400 and he complained of obstruction of his central vision. OCT showed the DHACM integrated into the inner retina with the outer retinal layers underneath (Fig. 1). Six months after the third surgery, we planned to remove his silicone oil and the DHACM. Of note, usually we prefer to remove the silicone oil within 3 months, however, this patient was unable to undergo another surgery sooner due to work. During vitrectomy for removal of DHCAM, the DHACM was noted to be adhered to the macula. A 25G Finesse Flex loop was used to initiate an edge of the graft. Maxgrip forceps were used to lift the available edge of the graft. Slow traction was applied as the graft peeled off the inner retina. There was careful observation during peeling for retinal tearing or re-opening of the macular hole. Following the removal of the DHACM, there was no observed retinal defect, hemorrhage, or reopening of the macular hole. Air fluid exchange was performed without gas or oil tamponade. At his 1 week post-operative visit, his vision improved to 20/80 with a flat retina, closed macular hole, and restoration of macular layers on OCT (Fig. 2). He was seen again 8 months after DHACM removal and the VA was stable at 20/80 at distance and J1+ at near, IOP 10. He reported resolution of the central scotoma and metamorphopsia and is pleased with the results.

**Fig. 3:**
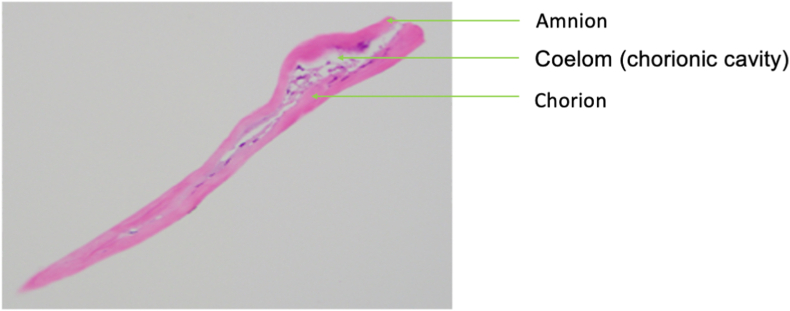
Excised fragment of amniotic tissue as provided by pathology lab.

In this case, the macular hole closure was successful using silicone oil endotamponade and DHACM. 6 months following the operation the macular hole remained closed. The amniotic membrane graft was removed along with the silicone oil due to the graft obstructing the fovea. The amniotic membrane graft was successfully removed (as presented in Video 1), and the tissue was submitted to the pathology team for evaluation of retinal integration. After 6 months of implantation, the 0.1cm fragment of hypocellular membranous amniotic tissue (Fig. 3) did not show obvious retinal tissue integration on multiple H&E-stained levels examined under high-magnification. Although OCT imaging prior to removal noted signs of the hAM integrating into the inner retina, there was no evidence on histopathology.

## 3 Discussion

The standard treatment of macular holes is pars plana vitrectomy (PPV) with internal limiting membrane (ILM) peel, gas endotamponade, and face down positioning. The primary closure rates with these techniques are reported to be greater than 90 %.^6^ However, even in the setting of a high success rate, recurrent or persistent macular hole treatment can be challenging. Standard techniques as described above are not as effective in the case of failed PPV and membrane peel. As multiple solutions have been suggested for the treatment of persistent macular holes, particularly over the last few years, there is no consensus regarding any appropriate selection criteria or the best surgical option. Modern approaches are associated with closing a full thickness macular hole using an alternative tissue to promote the anatomical closure and healing of these holes.^2^, ^5^ Some adjuvant materials and strategies have been used to increase the closing rate of refractory macular hole including internal limiting membrane (ILM) flap, autologous platelets, amniotic membrane graft, autologous retinal transplant. Of these notable modern approaches, human amniotic grafts have been proposed for their anti-inflammatory and antiangiogenic effects and as they secrete regenerative growth factors.^7^ It has been widely used in treating ocular surface problems since the 1940s. Human amniotic membranes were first attempted to repair retinal break and treat recurrent macular holes in 2018. We summarized several studies in Table 1. In that initial study, hAM (human amniotic membrane patches were used to close 8 recurrent macular holes with sulfur hexafluoride (SF6) used as the endotamponade. The anatomical success rate was reported to be 100 % at 6 month follow-up. Since then, there have been studies showing that hAM plugs can be effective in repairing retinal breaks, large macular hole associated with retinal detachment, and high myopic macular holes associated with retinal detachment. Zhang et al. presented the first systematic and meta-analysis of the efficacy and complications of hAM grafts in the management of refractory macular holes (MH) in 2023. ^3^In that study they reviewed 210 relevant articles and analyzed 8 total studies (comprised of retrospective and prospective articles). 8 studies were included in the single-arm meta-analysis with a total of 103 patients (103 eyes) who received hAM grafts. A total of 103 eyes that had MH were reported as closed in all 8 studies. The success rate in that study was 94 %, and the VA improvement rate was 66 % using a random effect model. 7 studies with 96 eyes included in that systemic and meta analysis reported the hAM graft dislocation/contracture rate which was 6 %. In that study they observed a difference in the macular hole closure rate and graft dislocation/contracture rate in patients using different types of hAM grafts. In the studies with cryopreserved hAM grafts, the macular hole closure rate was 99 % and the hAM graft dislocation/contracture rate was 3 %. In the studies using dehydrated hAM grafts, the macular hole closure rate was 73 % and the hAM graft dislocation/contracture rate was 26 %.^3^ It is worth mentioning that in the articles included in this systemic review, no intraocular inflammation was observed in all cases after hAM implantation. Only 1 document reported a mild increase of intraocular pressure, which led to the suggestion that amniotic grafts are a highly compatible intraocular graft. In addition, there were some initial reports of achieving 100 % MH closure rate in 14 patients using hAM without performing ILM peeling. In that report they indicated that hAM may help reduce surgical steps, however, no larger sample sized control studies verified this.

**Table 1:** Summarized table for studies mentioned in discussion.

Article	Type of Study	Conclusion	PMID	Authors
A Human Amniotic Membrane Plug to Promote Retinal Breaks Repair and Recurrent Macular Hoe Closure	Case Series (14 eyes)	Describes a new technique that used hAM plug in patients with persistent macular hole – achieved anatomical success in all cases.	30312261	Rizzo et al.^1^
DSAEK – Derived Glider Technique to Introduce Human Amniotic Membrane Patch through Small-Gauge Trocar for Retinal Pathologies	Case Series (8 eyes)	Eight surgical cases with difficult macular holes to assess the effectiveness of using smaller trocars, 25G, to reduce the stress to the hAM patch during insertion.	31849196	Caporossi et all^2^
Human amniotic membrane graft for refractory macular hole: A single-arm meta analysis and systematic review	Meta Analysis (8 studies included −103 eyes)	hAM in the treatment of the refractory MH resulted in visual improvement with MH closure rates. Cryopreserved hAM graft might have better outcomes than dehydrated grafts.	36739260	Zhang et al.^3^
Use of Epimacular Amniotic Membrane Graft in Cases of Recurrent Retinal Detachment Due to Failure of Myopic Macular Hole Closure	Case Series (14 eyes)	Epimacular hAM for myopic macular hole – retinal detachment is a safe and effective treatment for closure in patients with myopia.	32084283	Moharrem et al. ^4^
Human Amniotic Membrane Plug for Macular Holes Coexisting with Rhegmatogenous Retinal Detachment	Case Series (14 eyes)	Use of human amniotic membrane is a valid option in surgery for macular holes coexisting with rhegmatogenous retinal detachment. All patients had continued closure at six months.	32904754	Abouhussein et al.^5^

The first systematic and meta-analysis of the efficacy and complications of hAM grafts in the management of refractory MH, by Zhang et al., concluded that membrane grafts in macular hole surgery have very low intraoperation and post-operation complications and lead to improved postoperative best corrected visual acuity (BCVA).^3^, ^4^, ^8^ Furthermore, cryopreserved hAM grafts may have higher macular hole closure rate and lower grafts dislocation/contracture rate than dehydrated hAM grafts. Zhang et al. reported that MH closure rates were 99 % in the cryopreserved hAM group and 73 % in the dehydrated group. In addition, there were differences in the dislocation/contracture rate (3 % in the cryopreserved hAM group and 26 % in the dehydrated hAM group). The differences in the contracture and dislocation indicate that the differences in the scaffold properties of the cryopreserved and dehydrated graft may affect the efficacy of macular hole closure. Cryopreserved grafts tend to be more transparent and have more stroma, but difficult to flatten intraoperatively on a macular hole. In 2023 Chen et al. reported the results of macular hole repair in 12 eyes with autologous blood clot - assisted lyophilized hAM and 10 out of 12 eyes had successful closure of MH.^9^ Dehydrated grafts may provide better frame and structure for the retinal tissue to heal together, but can be less transparent and visually impairing, as in our case. How different graft types affect the success of surgery and the indications for certain cases remains to be further studied.

In this case, we used a dehydrated hAM graft with silicone oil endotamponade instead of gas – it is suggested that oil endotamponade may be easier for patients due to less restrictions on positioning. However, there is no data to suggest that there is a difference in the setting of macular hole repair using gas or oil endotamponade. Caporossi et al. performed a case series to assess the effectiveness of the human amniotic membrane (hAM) to treat high myopic macular holes (HMMH) associated with retinal detachment (RD). In their patient population of 10 patients, the initial 5 patients had silicone oil tamponades, and the remaining 5 patients with 10 % octafluoropropane (C3F8) gas. They removed the SO in all five patients 2 months following the procedure. They concluded hAM plug is an efficient substrate to manage HMMH associated with RD resulting in encouraging visual acuity recovery. No differences were mentioned between the gas and oil endotamponade.

The use of amniotic membrane grafting has been used as a supportive procedure across surgical subspecialties including ophthalmologists, dentists, urologists, burn specialists, ear, nose, and throat surgeons, gynecologists.^10^ Amniotic membrane grafts expresses anti-inflammatory, anti-microbial, and anti-fibrotic factors that are the attributable cause for the production of growth factors and appropriate healing. It is assumed that this accommodating quality is why amniotic membranes are so well tolerated. In this case following 6 months of implantation, we found that there was no retinal tissue attachment on the removed amniotic membrane graft (Fig. 3). As this was the first reported case, we are unsure if retinal integration would've formed with prolonged implantation. Rizzo et al.’s case series concluded that following hAM implantation, OCT scans demonstrated the macular hole closure without glial process but with a fully stratified retinal layer over the hAM patch in all their cases. During the follow-up period, the neuroretina over the hAM plug differentiated to form retinal layers, in particular in the outer layer such as the external limiting membrane.^1^ Rizzo et al.’s findings show that placing the graft beneath the retina, rather than over it as in our case, may also have appropriate integration with the retina on OCT imaging. The macular hole in this case report remained closed on follow-up with OCT confirming restoration of retinal layers.

## 4 Conclusions

In summary, this is the first case that shows the possibility and safety of removing a human amniotic membrane graft (hAM) after closure of a macular hole. In this specific case, a hAM graft with silicone oil tamponade was performed to repair a persistent full thickness macular hole refractory to pars plana vitrectomy with internal limiting membrane peel. The graft used in this case was a dehydrated graft. The hAM patch was removed after 6 months of placement as the patch was impeding central vision. Although OCT imaging, prior to hAM removal, noted the hAM integration with the inner retina, but there was no difficulty in removing the graft and no damage to retina was found. The macular hole remains closed with restoration of retinal layers. Patient experienced improvement in visual acuity and the blockage of his central vision resolved. The mechanism of action for human amniotic membrane grafts for the treatment of retinal injuries is not completely understood, but likely attributable to their anti-inflammatory and antiangiogenic properties.

## CRediT authorship contribution statement

**Hares Patel:** Writing – review & editing, Writing – original draft, Formal analysis, Data curation, Conceptualization. **Kayla Swiatek:** Writing – review & editing, Supervision, Conceptualization. **Marie Rivera-Zengotita:** Writing – review & editing, Investigation, Formal analysis, Data curation. **Jinghua Chen:** Writing – review & editing, Visualization, Validation, Supervision, Software, Resources, Project administration, Methodology, Investigation, Funding acquisition, Formal analysis, Data curation, Conceptualization.

## Patient consent

Patient consent obtained prior to publications of images. Please see attached document.

## Authorship

All authors attest that they meet the current ICMJE criteria for Authorship.

## Funding

No funding or grant support

## Declaration of competing interest

The authors declare that they have no known competing financial interests or personal relationships that could have appeared to influence the work reported in this paper.
